# Modification of Fear Memory by Pharmacological and Behavioural Interventions during Reconsolidation

**DOI:** 10.1371/journal.pone.0161044

**Published:** 2016-08-18

**Authors:** Janine Thome, Georgia Koppe, Sophie Hauschild, Lisa Liebke, Christian Schmahl, Stefanie Lis, Martin Bohus

**Affiliations:** 1 Institute of Psychiatric and Psychosomatic Psychotherapy, Central Institute of Mental Health, Medical Faculty Mannheim, University Heidelberg, Mannheim, Germany; 2 Clinic for Psychosomatic Medicine and Psychotherapy, Central Institute of Mental Health, Medical Faculty Mannheim, University Heidelberg, Mannheim, Germany; 3 Department of Theoretical Neuroscience, Central Institute of Mental Health, Medical Faculty Mannheim, University Heidelberg, Mannheim, Germany; 4 Department of Psychiatry, Schulich School of Medicine and Dentistry, Western University, London, Ontario, Canada; 5 Faculty of Health, University of Antwerp, Antwerp, Belgium; Western University of Health Sciences, UNITED STATES

## Abstract

**Background:**

Dysfunctional fear responses play a central role in many mental disorders. New insights in learning and memory suggest that pharmacological and behavioural interventions during the reconsolidation of reactivated fear memories may increase the efficacy of therapeutic interventions. It has been proposed that interventions applied during reconsolidation may modify the original fear memory, and thus prevent the spontaneous recovery and reinstatement of the fear response.

**Methods:**

We investigated whether pharmacological (propranolol) and behavioural (reappraisal, multisensory stimulation) interventions reduce fear memory, and prevent reinstatement of fear in comparison to a placebo control group. Eighty healthy female subjects underwent a differential fear conditioning procedure with three stimuli (CS). Two of these (CS+) were paired with an electric shock on day 1. On day 2, 20 subjects were pseudo-randomly assigned to either the propranolol or placebo condition, or underwent one of the two behavioural interventions after one of the two CS+ was reactivated. On day 3, all subjects underwent an extinction phase, followed by a reinstatement test. Dependent variables were US expectancy ratings, fear-potentiated startle, and skin conductance response.

**Results:**

Differential fear responses to the reactivated and non-reactivated CS+ were observed only in the propranolol condition. Here, the non-reactivated CS+ evoked stronger fear-potentiated startle-responses compared to the placebo group. None of the interventions prevented the return of the extinguished fear response after re-exposure to the unconditioned stimulus.

**Conclusions:**

Our data are in line with an increasing body of research stating that the occurrence of reconsolidation may be constrained by boundary conditions such as subtle differences in experimental manipulations and instructions. In conclusion, our findings do not support a beneficial effect in using reconsolidation processes to enhance effects of psychotherapeutic interventions. This implies that more research is required before therapeutic interventions may benefit from a combination with reconsolidation processes.

## Introduction

Fear is an important symptom in many mental disorders such as phobia, generalized anxiety disorder or posttraumatic stress disorder [1]. In a recent concept paper, Lane and co-workers [2] emphasized reconsolidation as a central mechanism in treating fear-relevant disorders, irrespective of the psychotherapeutic orientation. The authors proposed that incorporating new emotional experiences into previously reactivated memories might attenuate or actually erase fear by reconsolidating a modified memory trace. The updating of a learned fear memory stands in contrast to the mechanism of extinction learning that forms the basis for exposure-based techniques [3–5]. Extinction learning represents ‘inhibitory learning’, i.e. the formation of a second memory trace, which competes as a ‘safety memory’ trace with the original fear memory trace [6–9]. Although extinction learning is a well-established therapeutic intervention, it is well known to every clinician that patients may re-experience fear after exposure therapy: the fear may spontaneously recover over time, or be evoked when exposed to a relevant cue in the same or within a new context [10–13]. This is explained by a temporary dominance of the fear over the safety memory.

Studies on behavioural and pharmacological interventions targeting fear memory have revealed promising results across species when applied after reactivation of fear memory during the time window of reconsolidation processes [14–22]. Particularly, the administration of the ß-adrenergic receptor antagonist propranolol has consistently been shown to erase fear responses [23–29]. Declarative memory, i.e. the explicit expectation of the occurrence of an aversive event, was not affected by this intervention. However, the fear-potentiated startle response, which involves amygdala engagement, was strongly attenuated during re-extinction and reinstatement tests up to one month later [25]. Studies on combining extinction with reactivation of fear memories suggested that a behavioural intervention might be similarly efficient as a pharmacological one [18, 30–34]. Behavioural interventions have particularly attenuated skin conductance responses that primarily reflect explicit learning about contingencies between conditioned stimuli and the aversive event [35, 36]. However, in contrast to propranolol administration, behavioural interventions were less efficient in erasing the emotional response as measured by fear-potentiated startle [24, 37, 38].

In sum, the engagement of reconsolidation processes may indeed be suited to improve therapy of fear-related disorders. This was recently supported by first studies in samples with clinical features [39, 40]. Nevertheless, a growing body of work has failed to replicate the original findings for both the pharmacological intervention [41] (see also [42]) as well as the behavioural intervention [24, 37, 38, 43]. This suggests that further studies in healthy volunteers by independent research groups are required before these approaches can be translated into clinical practice [38].

Dialectical Behaviour Therapy for Posttraumatic Stress Disorder (DBT-PTSD) has been established as an intervention for PTSD following childhood sexual abuse [44]. In recent years, exposure-based techniques have been combined with reappraisal and multimodal sensory stimulation to diminish the influence of dissociative symptoms, which often hamper learning in this clinical group (see skills-assisted exposure [44]). Reappraisal aims at focusing attention on the emotion-eliciting event, rather than avoiding it, while simultaneously providing new information about the fear-associated cue and thus neutralizing its emotional impact [45–47]. Multimodal sensory stimulation ranks among ‘grounding skills’ [44, 48, 49]. Thus, external sensory information such as haptic, acoustic and visual stimuli is used to block dissociative symptoms, i.e. the disruption and fragmentation of usually integrated functions of consciousness [50]. An enhanced integration of external and internal stimuli is assumed to promote memory encoding [51, 52].

The present study aimed at 1) replicating the erasure of fear memory by propranolol by an independent research group, and 2) testing whether established therapeutic approaches may benefit from a preceding reactivation of fear memory, to modify fear memory by induction of reconsolidation processes.

We hypothesized that propranolol administration as well as behavioural interventions, i.e. reappraisal and sensory stimulation, attenuate fear memory, restricted to the reactivated fear memory.

## Methods

### Participants

A total of 80 female participants participated in the study. Subjects were recruited via a call for participants on the website of the Central Institute of Mental Health, Mannheim as well as via flyers. Participants were informed about electric stimulation and potential drug administration. Subjects underwent a psychiatric and medical interview by a trained psychologist and physician to ensure mental and physical health. In case of any medical condition that contraindicated the intake of propranolol, subjects were excluded from further participation. The study was approved by the ethical committee of the Medical Faculty Mannheim/Heidelberg University. Written informed consent was obtained from each subject before participating in the study. Participants received a reimbursement for participation.

Subjects filled in self-report questionnaires, to assess trait and state anxiety (German version of the State-Trait Anxiety questionnaire, STAI; [53, 54]), spider (German version of the fear of spiders questionnaire, FAS; [55]) as well as snake anxiety (German version of the snake questionnaire, SNAQ; [56]). For further details on these questionnaires see S1 Appendix 1.1.

### Procedure

**Fig 1:**
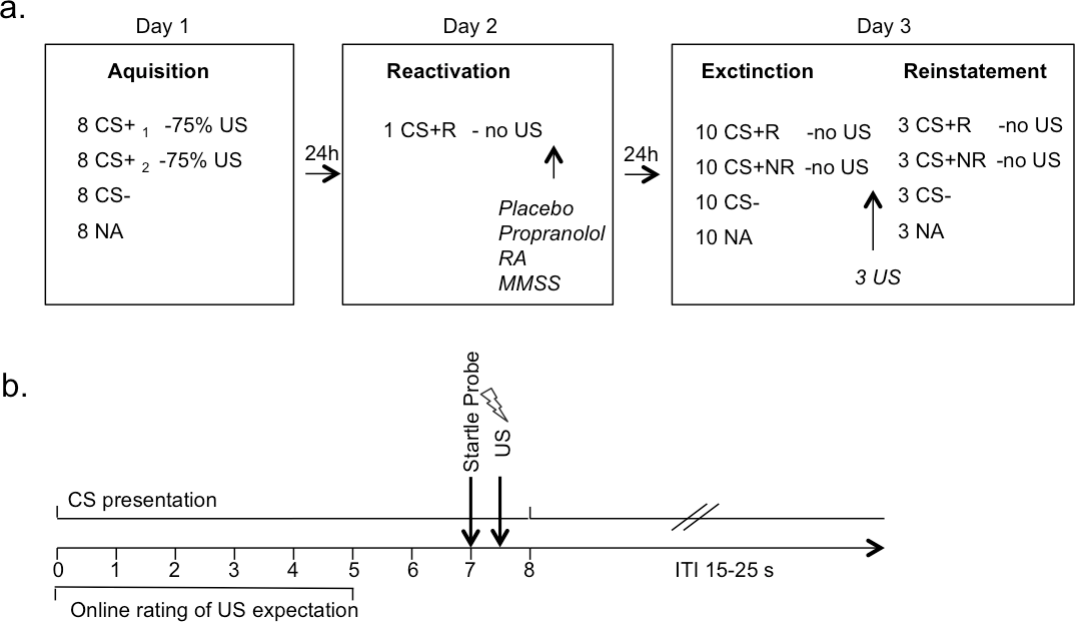
Details of the experimental design. a) Schematic illustration of the experimental design b) A conditioning trial of a reinforced stimulus presentation (stimulus duration 8s (within the first 7s US expectancy rating), startle probe 7s after CS onset, US 500ms after startle probe onset (electric shock for 2ms with an individually determined intensity).

The experimental procedure was adapted from the protocol by Kindt and co-workers (see [57]). On three consecutive days, participants underwent a fear acquisition phase (day 1), a memory reactivation phase combined with one of the four interventions (day 2), and a test phase during which retention, extinction and reinstatement of fear were assessed (day 3). See Fig 1 (for further information see S1 Fig).

#### Interventions during reconsolidation

During the reconsolidation time window, subjects underwent one of four interventions:

1. In the Propranolol condition, subjects received an oral dose of 40 mg propranolol similar as in previous studies (see [19, 23–29]).
2. In the Placebo condition, subjects received a placebo capsule, which was identical in size, shape and colour to the propranolol capsule. Both placebo and propranolol capsules were prepared and blinded by the pharmacy of the University Hospital Heidelberg.
3. In the Reappraisal (RA) condition, neutral information about the reactivated stimulus was presented for 15 minutes binaurally via headphones. For further details, see S1 Appendix 1.2.1.
4. The Multimodal Sensory Stimulation (MMSS) condition provided multiple sensory stimulation to each subject for 15 minutes: optic stimuli were applied by a fantasy movie trailer (e.g. “The Hobbit” by Peter Jackson, Warner Home Video; “Avatar” by James Cameron, Twentieth Century Fox) projected on a 150 x 150 cm (Lavolta, HD) screen (distance 270 cm); haptic stimulation by an massage chair providing intensive massage of the back area, and acoustic stimulation by a sample of daily noises (e.g. “Voice of America part 3/ LEGS” by Fred Frith, Step across the border, RecRec Music) presented binaurally via headphones. For further details, see S1 Appendix 1.2.2.

Subjects were assigned randomly to the intervention conditions. Placebo and Propranolol were assigned in a double-blind design.

#### Experimental Procedure

At each of the three testing sessions, participants were seated in a darkened room 80 cm in front of a computer screen. Each session started with a 5-minute resting period during which subjects watched an animal movie (“Winged Migration”, by Jacques Cluzaud, Michel Debats, Studiocanal) to allow habituation to the testing situation. Each day, throughout testing a 70 dB(A) broadband white noise was used as background noise. Testing started with a presentation of 10 startle probes to reduce initial startle reactivity. On each day, subjects rated state of anxiety (STAI-S) [53, 54] before and after testing. Blood pressure was measured at the start of each session as well as 90 minutes post intervention at day 2 (sphygmomanometer SBC 23, Sanitas). Subjects evaluated the US at the end of the session on day 1 and 3 on an 11-point rating scale ranging from -5 (unpleasant) to 5 (pleasant).

Day 1: Acquisition Phase: Three types of visual stimuli were presented 8 times each. Two fear-relevant stimuli were used as CS+ (spider and snake, IAPS, numbers 1220 and 1052) [58]. CS+ were linked in 75% of trials to an unpleasant, but not painful electric stimulation (US, duration 2ms, intensity individually adjusted). A fear-irrelevant stimulus served as a CS- (mug, IAPS number 7009) [58] and was never associated with an aversive event. Each CS presentation was coupled with a startle probe (duration of CS presentation 8s, onset of startle probe 7.5s after CS onset). For reinforced CS+ trials, the startle probe was followed after 500ms by the presentation of the US. In addition, 8 startle probes were presented alone (noise alone, NA). Inter-trial intervals (ITI) varied between 15, 20 and, 25 s (mean 20 s) (see Fig 1; for detailed description of stimuli and US characteristics see S1 Appendix 1.3.1).

Experimental trials were presented in a pseudo-random order with counterbalancing the position of the CS and NA trials. In each sequence, the first and last presentation of both CS+ were unreinforced to prevent that the reminder trial on day 2 results in extinction learning [59].

Subjects were instructed to learn to predict which of the pictures will be followed by an electric shock. Before the start of the testing, they were told that two pictures would be followed by an electric stimulus (US) in most of the cases, whereas a third picture would never be followed by the US. Immediately after onset of each stimulus, subjects were asked to rate how strongly they expected that an US would occur. At the end of the acquisition phase, subjects were explicitly instructed to remember what they had learned to enhance retention of the CS-US contingency on the following days [60].

Day 2: Memory Reactivation Phase and Intervention: To ensure consolidation of the acquired fear memory, an interval of 24 hours was inserted. After electrode attachment, participants were instructed to remember what they had learned the day before, and told that the same pictures would be presented again. During testing, one of the CS+ (CS+R) was presented to reactivate fear memory. The presentation of the CS+R was followed by one NA trial. The non-reactivated CS+ (CS+NR) served as a fear-conditioned control stimulus on day 3, to test whether effects of the interventions depend on reactivation, i.e. whether they selectively affect the fear response to the CS+R, but not the CS+NR (see for similar design [24, 29]). The assignment of the two CS+ to the reactivation condition was counterbalanced.

One of the four interventions was administered 5 minutes post reactivation. To standardize the experimental setting, participants of the placebo and propranolol condition were sitting in front of a black computer screen for 15 minutes. Participants of the RA and MMSS condition were exposed to the specific intervention, respectively (for details see S1 Appendix 2.2 and 1.2). Afterwards, electrodes were detached and participants were seated in a waiting room for about 60 minutes. Blood pressure was measured for 90 min after the onset of the intervention.

Day 3: Test Phases: Twenty-four hours after memory reactivation, participants underwent extinction training and reinstatement testing. Participants were told that the same three pictures provided on day 1 would be presented again. During the extinction phase, CS+R, CS+NR and CS-, as well as NA trials were presented 10 times, respectively. Subsequently, fear memory was reinstated by presenting three unsignalled reminder shocks (time interval between the last trial of the extinction phase and the first US: 19 s). In the reinstatement test phase, 3 CS and NA each were presented in a pseudo-randomized order. The first trial started 15s after the last unsignalled US.

#### Measurement Parameters

Fear response was assessed by three dependent variables, i.e. US online expectancy ratings, fear potentiated startle amplitude and skin conductance response.

US online expectancy (OE-R): With each CS+ and CS- onset, subjects were asked to rate their expectancy of an electric shock (11 point-Likert scale, ranging from -5: sure no shock to +5: sure a shock). The rating scale was displayed on the bottom of the screen and choices were selected by moving a cursor with a pen on a graphical tablet (Wacom intuos5 touch pen tablet, (PTH 850 IT)). Responses had to be done within a 5 s interval after stimulus onset. Missing values were replaced by means of moving average interpolation within each subject, stimulus type and experimental phase, except for the first and the last trial of fear acquisition and extinction learning, the reactivation trial and reinstatement testing. Regarding the first and last trial of fear acquisition and extinction learning, missing values were replaced by means of nearest neighbour interpolation. Fear Potentiated Startle (FPS): The conditioned fear response was measured via the potentiation of the eye-blink startle reflex to a loud noise by electromyography (EMG) of the left orbicularis oculi muscle. The acoustic startle reflex is a specific measure of fear [61], and is modulated by the amygdala [62]. Two electrodes with a diameter of 13 mm each, filled with electrolyte gel (Synapse, Costumer Kinetics) were positioned approximately 1 cm under the pupil and 1 cm below the lateral canthus [63]. The eye-blink EMG activity was measured with an EMG amplifier (Varioport, Becker Meditec, input resistance of 500 MΩ, bandwidth of 19–500 Hz (-3dB), sampling rate 1024Hz). EMG data were pre-processed using in-house software (MatLab 2011b, MathWorks) following a standard procedure [63]. Raw data were filtered (50Hz notch filter, 28-Hz high-pass filter, 4th order Butterworth filter), rectified and smoothed (low-pass filter 50Hz). Startle amplitude was measured as the maximum peak within a time window of 20–150ms following the onset of the startle probe referenced to mean baseline level (500ms before onset of the startle probe). Outliers were defined (Z > 3) and replaced by the maximum z-score. Missing values were replaced by means of nearest-neighbour interpolation within each subject, stimulus type and experimental phase, except the reactivation trial. To normalize the data and to reduce the influence of between-subjects variability, startle amplitudes across all phases of the study were standardized together using within-subject t-score conversion [38, 42]. Skin Conductance Response (SCR): Electrodermal activity was measured using an electrodermal response amplifier (Varioport, Becker Meditec) by applying a DC voltage of 0.5 Volt. Data was collected with a bandwidth of 0–50 micro Siemens (μS) and a solution of 0.002 μS. Two electrodes with a diameter of 13 mm were attached to the ball of the thumb on the non-preferred hand. Analogous to Kindt and colleagues [23, 35, 37], SCR elicited by the CS was determined by taking the peak-to-baseline difference within the 1 to 7 s window following stimulus onset. A minimum response criterion was set to 0.02 μS and filtered with 1 Hz. All other responses were scored as zero and included in the analyses [25]. Raw SCR scores were square-root-transformed to normalize distributions [18, 30, 33, 38].

### Statistical Analysis

Sociodemographic variables (age, years of education), trait anxiety, spider and snake phobia characteristics and the intensity chosen for the electrical shock were analysed in separate one-factorial variance-analytical designs (ANOVA) with ‘type of intervention’ as between-subjects factor. To examine differences in state anxiety between intervention groups over the course of testing, a 4 x 3 x 2 repeated measure ANOVA (rmANOVA) was applied with the between-subjects factor ‘type of intervention’ (Placebo vs. Propranolol vs. RA vs. MMSS) and the within-subject factors ‘experimental phase’ (acquisition, reactivation and test phase, i.e. day 1, 2 and 3) and ‘time’ (pre- vs. post-testing). In addition, evaluation of the US over the course of testing was assessed with a 4 x 2 rmANOVA with the between-subjects factor ‘type of intervention’ and the within-subject factor ‘day’ (day 1 and day 3).

To ensure comparability with previous studies and simultaneously reduce the complexity of the statistical designs, we compared each of the three active interventions (propranolol, RA, MMSS) to the placebo control condition in separate analyses. The effect of the intervention group on systolic and diastolic blood pressure on day 2 was analysed with a 2 x 2 rmANOVA, with the between-subject factor ‘type of intervention’ (placebo vs. propranolol) and the within-subject factor ‘time’ (pre-testing day 2 vs. 90 min post intervention day 2).

Fear response was assessed by three dependent variables (OE-R, FPS, SCR) and analysed for fear acquisition, retention, extinction learning and reinstatement, respectively.

We run separate rmANOVAs with the between-subject factor ‘type of intervention’ (Placebo vs. Propranolol/ RA/ MMSS) and the within-subject factors ‘stimulus type’ (CS-, CS+R, CS+NR) and ‘time’. The factor ‘time’ was calculated as following a) for the acquisition of fear: the 8 trials of the acquisition phase on day 1 averaged over two consecutive trials resulting in a 2 x 3 x 4-design, b) for the retention of fear: the average of the last 2 trials of the acquisition phase on day 1 and the average of the first two trials of the extinction phase on day 3 resulting in a 2 x 3 x 2-design, c) for extinction learning: the average of the first two trials and the last two trials of the extinction phase on day 3 (2 x 3 x 2-design), and d) for reinstatement: the average of the last two trials of the extinction phase and the first reinstatement test trial after the presentation of three reminder shocks on day 3 (2 x 3 x 2-design). For further description of statistical effects, post-hoc comparisons were calculated as appropriate by sub-designs of the main design or pairwise comparisons (Bonferroni adjusted for multiple testing).

Statistical significance was set to *p* < .05. All analyses were performed using SPSS (version 22; SPSS Inc., USA).

## Results

### Sample description

The four intervention groups were comparable in age (F(3,75) = .91, *p* = .442), years of education (F(3,75) = 1.39, *p* = .251), trait anxiety (F(3,75) = .83, *p* = .479), snake (F(3,75) = .89, *p* = .446) and spider phobia (F(3,75) = .94, *p* = .753). Moreover, intervention groups did not differ in their objectively selected electric shock intensity (F(3,75) = .97, *p* = .411) (see Table 1). US evaluation differed over time depending on the intervention (intervention x time: F(1,75) = 2.89, *p* = .041) with a reduction of unpleasantness over time for the propranolol, RA and MMSS groups compared to the placebo group. Consistent with other studies (e.g. [25]), intervention groups were comparable in their state anxiety over the course of testing (F(6,148) = 1.06, *p* = .389). For further details on sample characteristics see Table 1.

**Table 1:** Sample Description.

	Placebo	Propranolol	CRA	MMSS		
	(n = 19)	(n = 20)	(n = 20)	(n = 20)		
					*F*	*p*
age (*mean/SD)*	23.89 (3.06)	25.50 (3.71)	24.80 (6.15)	26.65 (7.42)	.97	.442
years of education *(mean/SD)*	11.78 (.63)	11.90 (.45)	11.60 (.82)	11.45 (.99)	1.13	.251
**US characteristics**				* *		
Shock Intenstity (mA) *(mean/SD)*	15.73 (8.67)	13.60 (7.27)	17.30 (7.21)	17.20 (8.14)	.97	.411
**Trait Anxiety Assessment**				* *	* *	* *
Spider Fear *(mean/SD)*	5.21 (8.21)	7.65 (8.49)	7.50 (11.19)	8.70 (12.26)	.40	.753
Snake Fear *(mean/SD)*	5.00 (5.02)	4.20 (3.16)	3.55 (2.94)	5.25 (3.14)	.89	.446
Trait Anxiety *(mean/SD)*	29.11 (5.09)	31.70 (5.85)	30.45 (5.78)	31.45 (5.94)	.83	.479
**US Evaluation**						
Day 1 *(mean/SD)*	-2.78 (1.68)	-3.50 (1.05)	-3.05 (1.76)	-3.30 (1.26)		
Day 3 *(mean/SD)*	-2.94 (1.22)	-2.45 (1.63)	-2.45 (1.79)	-2.80 (0.95)		
**State Anxiety Assessment**				* *		
day 1: state anxiety pre	29.16 (4.72)	32.52 (6.26)	31.70 (4.69)	32.50 (6.27)		
day 1: state anxiety post	31.63 (10.05)	35.35 (8.91)	33.20 (7.73)	32.40 (6.56)		
day 2: state anxiety pre	30.05 (6.28)	32.70 (8.19)	30.40 (5.24)	32.00 (6.74)		
day 2: state anxiety post	28.52 (4.71)	31.80 (6.21)	28.25 (6.27)	30.60 (6.23)		
day 3: state anxiety pre	29.62 (7.04)	34.15 (8.79)	31.60 (7.71)	31.35 (7.82)		
day 3: state anxiety post	30.42 (7.17)	33.75 (7.06)	31.85 (9.41)	33.70 (8.37)		

### Manipulation check propranolol

Systolic or diastolic BP as well as HR did not decrease in the propranolol as compared to the placebo group on day 2 (systolic BP: F(1,37) = 2.02, *p* = .164, diastolic BP: F(1,37) = .024, *p* = .878, HR: F(1,36) = 2.78, *p* = .102). For means and SD see S1 Table.

### Experimental tasks

**Fig 2:**
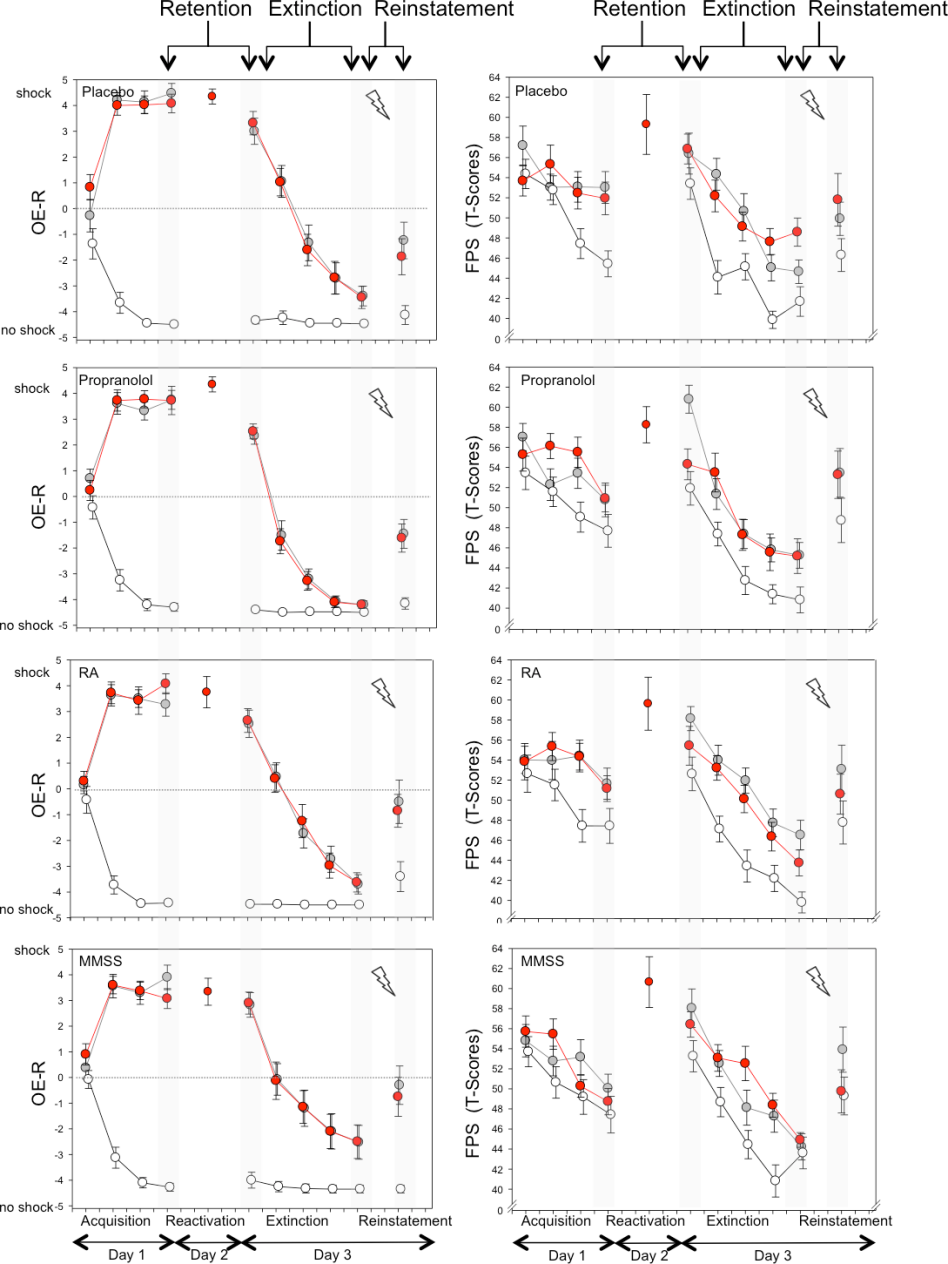
US online expectancy ratings and fear potentialed startle during acquisition, reactivation, and reinstatement testing. US online expectancy ratings (OE-R, left column) and fear potentiated startle (FPS, right column) for the four intervention conditions (placebo, propranolol, cognitive reappraisal (RA), multisensory stimulation (MMSS)) and the three types of stimuli (white circles: CS-, red circles: reactivated CS+, grey circles: non reactivated CS+) for the different phases of the experimental procedure (please note, data corresponds to averages across two consecutive trials, except for reinstatement, which displays the first trial after reinstatement)

Results of the ANOVAs are reported in Tables 2 and 3. Mean US expectancy ratings and fear potential startle amplitudes are displayed for the four intervention groups in Fig 2. Since statistical analyses revealed no differences for the behavioural interventions (RA, MMSS) as compared to the placebo condition (see Tables 2 and 3), we restrict the results description to the analyses of propranolol as compared to the placebo condition. For further information on the analyses for RA and MMSS, see S1 Appendix 2.1–2.4.

**Table 2:** Summary of rm ANOVAs testing US-E separate for each experimental phase. Intervention groups are compared to placebo condition, respectively.

	Propranolol		CRA		MMSS	
	F	p	F	P	F	p
**Acquisition**						
Intervention	.14	.712	.49	.484	.15	.698
Stimulus Type	462.74	< .001*	548.04	< .001*	446.46	< .001*
Time	15.544	< .001*	18.58	< .001*	13.89	< .001*
Intervention x Stimulus Type	1.78	.175	1.46	.240	3.12
Intervention x Time	1.05	.372	1.98	.121	2.64	.053*
Stimulus Type x Time	67.44	< .001*	77.51	< .001*	68.56	< .001*
Intervention x Stimulus Type x Time	1.10	.362	1.018	.414	.21	.98
**Retention**									
Intervention	2.60	.115	1.67	.205	.89	.350
Stimulus Type	707.74	< .001*	576.81	< .001*	483.28	< .001*
Time	12.65	< .001*	13.69	.001*	5.76	.022*
Intervention x Stimulus Type	1.46	.239	1.16	.318	1.55	.219
Intervention x Time	.21	.647	.02	.901	.75	.391
Stimulus Type x Time	7.87	.001*	7.24	.001*	8.15	.001*
Intervention x Stimulus Type x Time	.24	.792	2.03	.139	.21	.815
**Reinstatement**						
Intervention	.24	.627	.33	.567	1.29	.262
Stimulus Type	29.68	< .001*	18.98	< .001*	31.87	< .001*
Time	42.14	< .001*	55.22	< .001*	27.88	< .001*
Intervention x Stimulus Type	.23	.794	.08	.920	1.52	.23
Intervention x Time	1.60	.214	4.66	.038*	.01	.946
Stimulus Type x Time	15.31	< .001*	6.54	.002*	10.65	< .001*
Intervention x Stimulus Type x Time	.78	.462	.094	.910	.18	.837

**Table 3:** Summary of rm ANOVAs testing FPS separate for each experimental phase. Intervention groups are compared to placebo condition, respectively.

	Propranolol		CRA		MMSS	
	F	p	F	p	F	p
**Acquisition**						
Intervention	.08	.781	.03	.876	.37	.549
Stimulus Type	13.69	< .001*	14.21	< .001*	12.56	< .001*
Time	12.73	< .001*	8.89	< .001*	14.48	< .001*
Intervention x Stimulus Type	.67	.515	.15	.858	.68	.508
Intervention x Time	.59	.623	.70	.553	.190	.903
Stimulus Type x Time	3.05	.007*	2.99	.008*	1.88	.085
Intervention x Stimulus Type x Time	.65	.691	.72	.637	1.53	.169
**Retention**									
Intervention	.02	.889	.02	.892	.38	.542
Stimulus Type	14.79	< .001*	12.89	< .001*	9.71	< .001*
Time	20.22	< .001*	15.94	< .001*	24.97	< .001*
Intervention x Stimulus Type	.99	.377	.35	.705	.81	.448
Intervention x Time	.03	.854	.001	.970	.48	.492
Stimulus Type x Time	.88	.418	.62	.538	.21	.814
Intervention x Stimulus Type x Time	3.77	.028*	1.24	.297	1.68	.194
**Reinstatement**									
Intervention	.63	.433	.04	.841	.15	.703
Stimulus Type	11.97	< .001*	10.88	< .001*	4.46	.015*
Time	22.48	< .001*	24.47	< .001*	21.49	< .001*
Intervention x Stimulus Type	.62	.539	2.34	.103	2.26	.111
Intervention x Time	2.28	.140	1.36	.252	.94	.340
Stimulus Type x Time	.148	.862	.25	.776	2.05	.136
Intervention x Stimulus Type x Time	.24	.786	.23	.793	.34	.713

### Fear Acquisition Propranolol compared to Placebo

To test for the successful acquisition of fear, we analysed the change of the dependent variables over the course of the acquisition phase. Differences between both CS+ compared to the CS- in the linear trend as well as increased responses to both CS+ as compared to the CS- at the end of fear acquisition phase are indicative of successful fear acquisition.

US Online Expectancy Ratings (OE-R): OE-R differed between stimulus types depending on time (F(6,222) = 67.43, *p* < .001; see Table 2, Fig 2). This effect was not influenced by the intervention condition (intervention x stimulus x time: F(6,222) = 1.10, *p* = .362). The linear trend over time differed for both the CS+R and the CS+NR compared to the CS- (post-hoc contrasts: CS+R: F(1,36) = 100.69, *p* < .001; CS+NR: F(1,36) = 191.92, *p* < .001). A direct comparison of the OE-R between CS+R and CS+NR revealed no differential change over time (2x4-ANOVA sub-design: stimulus x time: F(3,114) = .71, *p* = .546). Post-hoc tests revealed that at the end of the acquisition phase the OE-R was higher for CS+R and CS+NR as compared to CS- (both *p* < .001), while no differences were found between both CS+ (*p* > .1).

Fear Potentiated Startle (FPS): FPS differed between stimulus types depending on time (F(6,216) = 3.05, *p* = .007; see Table 3, Fig 2). This effect was not influenced by the type of intervention (intervention x stimulus x time: F(6,216) = 0.65, *p* = .663). The linear trend over time differed for both the CS+R and the CS+NR compared to the CS- (post-hoc contrasts: CS+R: F(1,36) = 5.71, *p* = .022; CS+NR: F(1,36) = 4.59, *p* = .039). In addition, the change in FPS change over time was different between CS+R and CS+NR (2x4-ANOVA sub-design: stimulus x time: F(3,111) = 3.03, *p* = .033). While there were no differences in the linear trend between both conditions (F(1,36) = 0.41, *p* = .527), post-hoc contrasts revealed a differential quadratic trend (F(1,36) = 7.01, *p* = .012). This suggests that the interaction effect was caused by a stronger change in CS+NR as compared to the CS+R at the start of the acquisition phase. However, at the end of the acquisition phase FPS was higher for CS+R and CS+NR as compared to CS- (both *p* < .003) and there was no difference between both CS+ (*p* > .1).

Skin Conductance Response: Overall analysis of variance did not reveal significant fear acquisition (stimulus x time: all F’s < 1.81, all *p’s* > .099). Therefore, no further data on SCR are reported.

### Retention Propranolol compared to Placebo

The retention of fear is measured as the strength of the fear response at the start of the extinction phase compared to the end of the acquisition phase. A stronger reduction of fear in response to the CS+R as compared to the CS+NR and a lower fear response to the CS+R compared to the CS+NR at the start of extinction, indicate a fear attenuation effect, which is specific for the reactivated CS+.

US Expectancy Ratings (OE-R): OE-R differed between stimulus types depending on time (F(2,74) = 7.86, *p =* .001; see Table 2, Fig 2) without an influence of the intervention condition (intervention x stimulus x time: F(2,74) = .24, *p* = .792). The OE-R change from the end of the acquisition phase to the start of extinction differed for both the CS+R and the CS+NR as compared to the CS- (post-hoc contrasts: all *p*’s < .015). No difference was found in OE-R between CS+R and CS+NR depending on time (2x2-ANOVA sub-design: stimulus x time: F(1,38) = 1.45, *p* = .236). Post-hoc tests revealed that OE-R dropped for both CS+ from the end of acquisition to the start of the extinction phase (both *p* < .05) while there was no change in O-ER for CS- (*p* = .780). Nevertheless, OE-R was higher in response to the CS+R and the CS+NR compared to the CS- (both *p* < .001) without a difference between OE-R of both CS+ at the start of the extinction phase (*p* > .1).

Fear Potentiated Startle (FPS): FPS differed between the two intervention conditions depending on stimulus type and time (intervention x stimulus x time: F(2,72) = 3.77, *p* = .028; see Table 3, Fig 2). Intervention conditions differed in change of FPS over time when comparing the CS+NR to the CS- (F(1,36) = 6.39, *p* < .016), but not when comparing the CS+R to the CS- (F(1,36) = 0.36, *p* = .551). A direct comparison of both CS+ with an additional ANOVA sub-design revealed that intervention conditions differed in FPS change also on a trend-level for CS+R and CS+NR (2x2x2-ANOVA sub-design: intervention x stimulus x time: F(1,36) = 3.94, *p* = .055). At the start of the extinction phase, FPS was higher in response to the CS+NR as compared to the CS- as well as to the CS+R in the propranolol condition (both *p* < .011), while FPS did not differ between the three stimulus types in the placebo condition (all *p* > .411).

### Extinction learning Propranolol compared to Placebo

To test effects during extinction learning, dependent variables were contrasted for the start to the end of the extinction phase.

US-Expectancy Ratings (OE-R): OE-R differed between stimulus types depending on time (F(2,74) = 267.51, *p* < .001; see Table 2, Fig 2). This effect was not influenced by the intervention condition (intervention x stimulus x time: F(2,74) = .06, *p* = .944). The OE-R change from the start to the end of the extinction phase differed for both the CS+R and the CS+NR as compared to the CS- (post-hoc contrasts: all *p*’s < .001). No difference was detected in OE-R between CS+R and CS+NR depending on time (2 x 2-ANOVA sub-design: stimulus x time: F(1,38) = .77, *p* = .386). Post-hoc tests revealed lower OE-R to both CS+ during the end as compared to the start of extinction learning (both *p* < .001), while only a trend was found for CS- between phases (*p* = .086). At the end of the extinction phase, OE-R was still higher for both CS+ compared to the CS- (both *p* < .013), while there was no difference between both CS+ (*p* > .1).

Fear potentiated startle (FPS):. FPS differed between stimulus types depending on time without an influence of the intervention condition (stimulus x time: F(2,68) = 3.37, *p* = .040; intervention x stimulus x time: F(2,68) = .64, *p* = .533; see Table 3, Fig 2). While no differential change over time was revealed by post-hoc contrasts for both CS+ compared to the CS- (both *p*’s > .114), a direct comparison between both CS+ revealed a stronger decrease of FPS from the start to the end of the extinction phase for CS+NR than for CS+R (2x2-ANOVA sub-design: stimulus x time: F(1,36) = 7.04, *p* = .011).

### Reinstatement Propranolol compared to Placebo

The reinstatement of fear is measured as the strength of the fear response at the end of the extinction phase compared to the first trial upon the unsignalled US presentations. A stronger increase of fear in response to the CS+NR as compared to the CS+R and a lower fear response to the CS+R compared to the CS+NR in response to the reinstatement test trial, indicate the prevention of fear memory recovery, which is hypothesized to be specific for the reactivated CS+.

US Expectancy Ratings (OE-R): OE-R differed between stimulus types depending on time (F(2,70) = 15.31, *p* < .001; see Table 2, Fig 2). This effect was not influenced by the intervention condition (intervention x stimulus x time: F(2,70) = .78, *p* = .462). The OE-R change from the end of the extinction phase to reinstatement differed for both the CS+R and the CS+NR as compared to the CS- (post-hoc contrasts: all *p*’s < .002). No difference was found in OE-R change between CS+R and CS+NR (2x4-ANOVA sub-design: stimulus x time: F(1,36) = 2.22, *p* = .145). Post-hoc tests revealed higher OE-R to both CS+ at reinstatement testing as compared to the end of extinction learning (both *p* < .001), while change over time regarding the CS- was only a trend (F(1,36) = 3.25, *p* = .094). At reinstatement testing, OE-R was higher to both CS+ compared to the CS- (both *p* < .020), with no difference between both CS+ (*p* > .1).

Fear Potentiated Startle (FPS): FPS increased over time (F(1,34) = 22.49, *p* < .001; see Table 3, Fig 2), independently of the intervention condition and the stimulus type (intervention x stimulus x time: F(2,68) = .24, *p* = .786; stimulus x time: F(2,68) = .15, *p* = .829). In general, FPS differed between stimulus types (F(2,68) = 11.97, *p* < .001). Post-hoc tests revealed a larger FPS to both CS+ as compared to the CS- overall (both *p* < .001), with no differences between CS+ (*p* > .1).

## Discussion

The objective of the present study was to investigate whether different interventions are suited to alter or erase fear memory by targeting reconsolidation processes. We aimed at a) replicating the fear-erasing effect of propranolol in disrupting reconsolidation processes, and b) testing effects of reappraisal and sensory stimulation on fear memory, when applied after reactivation of a fear-associated stimulus during the time window of reconsolidation. As expected, during retention testing we observed 1) comparable fear responses for the reactivated fear-conditioned stimulus and the non-fear associated stimulus and 2) stronger fear responses to the non-reactivated fear-conditioned stimulus. However, these effects could not be explained by an erasure of fear for the reactivated CS+, but instead by a stronger fear response to the non-reactivated CS+ as revealed by the placebo control condition. Thus, we were not able to replicate an erasure of fear by the administration of propranolol. In contrast to our hypotheses, RA and MMSS did not result in an attenuation of fear for the reactivated fear-conditioned stimulus. Instead, we observed a similar fear response during retention and reinstatement testing for all stimulus types, suggesting a generalization of fear to the neutral control stimulus.

Several studies by Kindt and co-workers have demonstrated a beneficial effect of propranolol on fear memory. This led to increasing hopes for a successful translation of this approach to the treatment of fear in mental disorders [23–29]. A precondition for this effect is the reactivation of the fear-associated stimulus: In the studies by Kindt and co-workers, fear was selectively reduced for the reactivated CS+, but not for the non-reactivated CS+ suggesting a potential specificity for targeting pathological fear responses [24, 29]. Semantic instead of perceptual differences between fear-associated stimuli appear to be essential to trigger differential disruption of reconsolidation processes [19]. In variations of their experimental setups, Kindt and co-workers consistently demonstrated that propranolol reduced fear-potentiated startle responses when the substance was effective during the time window of reconsolidation, i.e. when propranolol was applied between 90 min before [23–25, 29] to 5 min after reactivation [26–29]. Beneficial effects were observed both during retention and reinstatement testing for FPS, as well as during a follow-up one month later, suggesting that fear memory was not only attenuated but also actually erased [25]. These effects were observed for FPS. In contrast, contingency learning, i.e. measurements of declarative memory such as online ratings of the expectation of the aversive event, as well as skin conductance response were not influenced by a propranolol administration [23, 25, 27, 28, 41, 42]. One explanation for these differential effects on emotional and declarative fear memory is that propranolol blocks ß-adrenergic receptors within the basolateral amygdala, which are needed for long-term potentiation and thus specifically disrupts the emotional expression of fear [23, 25, 64–66]. In line with these studies, we found no effects of propranolol administration on the cognitive level of fear memory, i.e. in online expectancy ratings, but differences in the emotional fear response during retention testing, depending on a reactivation of the fear-associated stimulus. Retention of fear measured by FPS was stronger for the non-reactivated CS+ as compared to the CS- and the reactivated CS+. Contrasting these changes with those observed after placebo administration revealed that fear response did not differ for the CS- and the reactivated CS+, but only for the non-reactivated CS+. This suggests that differences between stimulus types were not actually explained by an erasure of fear towards the reactivated CS+, but by a stronger fear response to the non-reactivated CS+. In line, reinstatement testing did not reveal a differential effect of propranolol upon the different stimulus types suggesting that indeed fear was not erased but generalized to the previously neutral CS-.

Our findings are in line with previous studies that failed to replicate beneficial effects of a pharmacological or behavioural intervention during the time window of reconsolidation. A recent study of the research group of Kindt could not replicate the otherwise often demonstrated erasure of fear after propranolol administration without identifying the conditions that may have prevented the beneficial effect [41]. The present study, which similarly found no fear-erasing effect of propranolol, was to the best of our knowledge the first attempt to replicate the effects of propranolol by an independent research group. In contrast to the pharmacological intervention with propranolol, there exist several studies by independent groups that aimed at replicating the effects of behavioural interventions as first described by Schiller and co-workers [30]. Some of these confirmed a superior fear attenuation of extinction when applied during reconsolidation [31, 32, 34], however, several others did not replicate this effect [24, 37, 38, 43].

These inconsistent findings have recently been discussed in the context of so-called boundary conditions that may restrict the induction of reconsolidation processes, and as a consequence, the potential to actually modify a consolidated memory trace [1, 9, 38, 42].

Boundary conditions comprise e.g. features of the experimental procedures, which can affect the memory strength during acquisition and the updating of a memory trace. Moreover, characteristics of the memory itself such as its age as well as features of the enrolled participants may influence findings. However, none of these boundary conditions appear to be able to explain our propranolol findings. Regarding experimental features, we aimed to closely follow the experimental protocol of the studies of Kindt and co-workers [24, 29, 67]. For example, a higher number of CS-US pairings, as well as a higher US reinforcement rate have been linked to stronger fear memories, which are particularly resistant to modification during reconsolidation [34, 68, 69]. Both CS-US pairings as well as reinforcement rates in our study were comparable to those used in previous experiments (e.g. number of CS-US pairings = 6, similar to [23, 25]; reinforcement rate = 75%, similar to [23, 25]). Memory updating may also be hampered if the memory retrieval trial does not present novel or relevant information. Instead, the reminder trial has to generate a mismatch between what is expected and what actually happens [27, 28, 70]. However, the expectancy rating as well as the FPS in response to the reminder stimulus on day 2 indicated a high expectation of the US, as well as an emotional fear response, suggesting the occurrence of the mismatch required for memory updating (see Fig 2, S1 Appendix 2.5) [27, 28, 70]. Moreover, the age of the acquired fear memory is in line with previous studies, i.e. reactivation was done 24 h after fear acquisition [18, 23–25, 27, 28, 30, 70]. Finally, fear erasure is less efficient in subjects high in trait anxiety [71]. However, an additional analysis of the potential link between trait anxiety and retention of fear revealed no association, suggesting that trait anxiety does not contribute to our findings (see S1 Appendix 2.6).

Similarly to the failure of propranolol to erase fear memory, our data does not support beneficial effects of the combination of established therapeutic techniques and reconsolidation processes. Neither reappraisal nor multisensory stimulation resulted in an attenuation of fear, not to mention an erasure of fear. This was true for both contingency learning, as well as the emotional fear response measured by FPS. While the mentioned boundary conditions affect pharmacological as well as behavioural interventions, the choice of the selected stimulus material may have particularly contributed to our findings regarding reappraisal or multisensory stimulation. Most studies confirming a beneficial effect of behavioural interventions, i.e. extinction training during reconsolidation, associated the fear response to neutral stimuli such as coloured geometrical shapes [18, 30–34]. In contrast, we used fear-relevant stimuli paired with an electric shock (see for similar design [24, 29]) and these are assumed to be more resistant to extinction learning. Therefore, this experimental feature may have contributed to the missing effect of reappraisal or multisensory stimulation. However, Golkar and co-workers [38] targeted the fear-relevance of the applied stimuli and failed to replicate fear attenuation after a behavioural intervention with both fear-relevant and fear-irrelevant stimuli.

Irrespective of the intervention group, we observed a generalization of the emotional fear response to the CS- during both, retention and reinstatement testing. Although generalization of fear is observed in many studies [72–74] and is discussed as an important factor in the development of fear-related mental disorders [75], the exact underlying mechanism is still under debate [76]. Generalization of fear has been related to many different processes ranging from the perception of stimuli features [73, 77] over category induction, comprising pre-existing knowledge about the nature of a relationship [76], to new category learning [78]. Moreover, subject characteristics such as the level of trait anxiety or the affective mood modulate generalization processes [79]. In studies on pharmacological and behavioural interventions during reconsolidation, generalization of fear to the non-fear-conditioned control stimulus have been observed simultaneously with a failure to erase fear (e.g. [37, 38, 41, 42]). Golkar et al. [38] observed a generalization of fear during reinstatement in response to fear-relevant, but not to fear-irrelevant stimuli, while Kindt and colleagues [37] linked a generalization of fear during reinstatement to subjects’ trait anxiety. In line with that, worrying about feared outcomes resulted in an enhanced FPS to the CS- [80]. A recent study by Geschwind et al. [79] suggests that the affective state after learning modifies safety learning and the generalization of fear. Similarly, Onat & Büchel [81] emphasized the relevance of ambiguity-based uncertainty and threat identification processes when building a flexible and adaptive fear response.

Based on our data, we cannot identify factors responsible for the observed generalization effect. However, exploratory analyses of our data revealed that trait anxiety is not sufficient to explain the generalization of fear (see S1 Appendix 2.7). Overall, generalization is a complex phenomenon. Further studies are needed that investigate in detail which factors may contribute to generalization in standard procedures for studying modifications of fear memory in the context of reconsolidation processes.

Finally, some limitations of the present study have to be addressed. First of all, the physiological effect of propranolol was not confirmed by heart rate and blood pressure decreases. However, the mean BP of our sample points to a floor effect: Baseline BP level in our sample equalled that observed after propranolol administration in previous studies [25, 29, 42] (see S1 Table). Moreover, we included only female subjects. While this resulted in a highly homogenous sample, it limits the generalizability of the present findings. Since female participants were overrepresented in recent publications [23–26, 29], it appears to be unlikely that our findings can be explained by this sample characteristic. Nevertheless, further studies are required that investigate potential gender effects on the studied processes. Finally, in contrast to declarative memory, emotional fear learning was supported statistically only on a trend-level for the multisensory intervention condition. Thus, findings on this intervention have to be interpreted with caution. Nevertheless, we observed discrimination between stimulus types at the end of fear learning in FPS. In addition, FPS as well as US expectation in response to the reactivated conditioned stimulus on day 2 suggests that at least the reactivated CS+ evoked a fear response. Finally, we were not able to investigate potential effects of our interventions on SCR since this parameter did not reveal fear acquisition. While studies by Kindt and co-workers [23–26, 28, 29] suggest that propranolol administration had no effect on SCR, behavioural interventions have affected particularly this variable [18, 30–33]. While previous studies on behavioural interventions [18, 30, 33] have excluded up to one third of the studied sample to restrict analyses to only those subjects that exhibited higher SCR to CS+ compared to CS-, a similar procedure in the present study would have resulted in too small sample sizes to explore potential effects of reappraisal and sensory stimulation on SCR.

## Conclusion

Reconsolidation research points to an efficient mechanism, which could be used for the treatment of anxiety disorders. Indeed, first investigations revealed promising results [39, 40]: combining reactivation with a pharmacological agent in spider-fearful participants [39] or a visuo-spatial task in subjects exposed to experimentally induced “trauma” [40] attenuated fear memory and prevented fear-related behaviour. Yet, an increasing number of studies–including our experiments–suggest difficulties in triggering reconsolidation processes. Although many boundary conditions for inducing reconsolidation have been described in the past years, these are not sufficient to explain the failure of replications. Moreover, the age of fear memories, as well as a higher anxiety in clinical samples may hamper the use of reconsolidation processes in the treatment of mental disorders. Thus, further basic research is needed to deepen our understanding of reconsolidation processes, before this approach can efficiently be translated into the clinical practice of treating anxiety disorders.

## Supporting Information

S1 Appendix Additional information regarding the study design and results. (PDF)

S1 Fig Experimental Procedure. Detailed description of the experimental procedure over three consecutive days.
(TIF)

S1 Table Systolic, and diastolic blood pressure, and heart rate. Mean values (SD) of systolic and diastolic blood pressure (in mmHg) and HR pre and 90 minutes post propranolol administration during memory reactivation on day 2.
(PDF)
