# Supplementary material for: Modification of Fear Memory by Pharmacological and Behavioural Interventions during Reconsolidation
Source: PLoS One. 2016 Aug 18;11(8):e0161044. doi: 10.1371/journal.pone.0161044 (PMC4990323; doi:10.1371/journal.pone.0161044)
Supplement: S1 Appendix — (PDF) [file pone.0161044.s001.pdf]

Supporting Information

S1 Appendix. Additional information regarding the study design and results

1. Methods

1.1 Subjective Assessment

The STAI [1, 2] is a 40 item questionnaire, with 20 items assessing trait anxiety (STAI-T) and 20 items measuring state anxiety (STAI-S). Each item is rated on a 4-point intensity scale. The questionnaire is characterized by a high degree of internal reliability (STAI-S: Cronbachs alpha = .92; STAI-T: Cronbachs alpha = .90), and discriminant validity in differentiating between anxiety and non-anxiety disorder groups [3].

The FAS [4] is an 18 item self-report measurement, comprising spider-anxiety related items. Each item is rated on a 7 point likert scale ranging from 0 (not true) to 6 (true), with a maximum score of 108. The questionnaire reached excellent internal reliability (Cronbachs alpha = .96) and retest reliability (rtt = .95). Moreover, the instrument is proven to discriminate appropriately between subjects with and without spider phobia (non overlapping distributions: non spider anxiety 0.6, spider anxiety 15-92; mean spider anxiety = 58.7) [4].

The SNAQ [5] is a 30 items questionnaire to assess fear of snakes. Subjects are instructed to state whether each item is true or not, with true answers forming the sum score (0-30). The questionnaire has shown good test-retest variability (rtt = .78) and internal consistency (Cronbachs alpha = .89) [5]. However the discriminant validity seems to be controversial, since high SNAQ scores (> 13) do not necessarily reflect avoidance behavior [6].

1.2 Interventions during reconsolidation

1.2.1 Cognitive Reappraisal.

Depending on the reactivated CS+ on day 2, either a neutral narrative about spiders or snakes were presented binaurally via headphones.

Spider. Spiders are very important animals, which are home in all ecological systems. They contribute to biodiversity und take a regulatory function in the habitat. For some habitats, more than a million spiders per hectare were estimated, which eat around 50 tons of prey per year – or far more than one million animals. They mostly consume insects. Doing that, they pay an important contribution to the maintenance of the natural equilibrium. Their body consists of two parts. On the upper body they have two chelicerae, two little feelers, eight legs and mostly eight eyes. On the lower body, the spider warts are located, in which die spider silk is produced. Worldwide exist more than 38 000 kinds of spiders. Of these, only twenty are dangerous for humans. None of them lives in central Europe. In Germany live cerca 1004 different, but harmless kinds of spiders. One very interesting spider is the water spider: The water spider is the only spider, which lives undersea. How does she manage not to choke? Basically, she does is very similar to scuba drivers. She always has a supply of oxygen stored. If she needs oxygen, she sticks her abdomen out of the water and pulls it back under water very quickly. In doing so, little air bubbles get stuck in the hair of her abdomen and hold onto it. The water spider then brings the air bubbles to her self-yearned diving bell under water, whereby an oxygen store is created. She only rarely stays at the water surface. In her driving bell she does not only store oxygen – in that place she recovers, eats and watches her babys while hatching. Furthermore spiders are very useful animals. On one hand there are useful insect eaters. Aphids, moths, flies and other arthropods are welcome delicacies on their menu. Doing this, they contribute to the balance of the ecosystem. Thus the night active eight-legged animals are important for the agriculture. The spiders help

eliminating the pest in farming. Also in medicine, the poison of the spider finds application. Using a bird spider's poison, a new medicament against heart failure was developed. For many people, the spider's web is a fascinating artwork. They produce threads as safety rope, for building up cocoons, as ballooning and especially as catching device that means webs. The threads consist of protein, because of that the orb-weaver spider can eat her old web, when building a new one. The physical processes of the thread production are still not completely understood. But it is known, that the thread consists of liquid protein, in which protein crystals are stored. Spiders own a sortiment of spinneret, which produce threads with different qualities for different purposes. Depending on the purpose the thickness of the threads lays around a thousandth part of a millimeter. Many kinds of spiders besides produce special fine threads of a hundred-thousandth part of a millimeter. Interestingly, the spider threads are as tear-proof as nylon und simultaneous as double stretchable. Therefore, there were especially many efforts to use spiders' threads for the production of textiles. The undertaking failed basically because the spiders needed to be held alone and this was too costly. Today, there are trials intending to let bacteria or dwarf goats produce the spiders' silk by implanting the genome of spiders. Until now, they do produce the right protein, but the connection of the threads was not accomplished yet. Furthermore, spiders are mostly loner. Although there are a series of exceptions: freshly hatched spiders stay a few days or weeks with their siblings together. In some species they are guarded by the mother, wolf spiders carry their babies on their back und some species even feed them with predigested food. Another species of spiders of a subtropical kind builds a catching web together and share their prey.

Snake. The evolution of the snakes begun many million years ago, a lot earlier than the evolution of the humans. Fossil discoveries from cretaceous age from around 95-100 million years ago indicate that. Thus, Snakes are real survival fighters. For example they can get along until 24 month without food, by strongly downregulating their metabolism. Snakes have

the ability to feel vibrations on the ground very sensitively to protect them from enemies, compensating their blindness. Snakes do have a nose, with which they are able to smell, but their tongue supports them regarding odors closely to the ground. How do they do that? They pick up little odorous substances with their tongue and transport them to their mouth. In the front of the snake's palate there are recesses – the so called Jacobson-Organ. Exactly here odorous substances are processed and therefore can be perceived. The perception works very similar to the perception in the nose. Although the Jacobson-Organ is a lot more sensitive. Furthermore snakes belong to the cold-blooded animals. That means that they cannot regulate their body temperature by using their metabolism. But how do they regulate their temperature instead? They do this, rolling up or moving to a cooler place, that means they use the outdoor temperature to compensate their body temperature. Because of that, it is possible that snakes fall into torpidity at lower temperatures. This can already happen at outdoor temperatures from 1°C to 9°C. To prevent torpidity or to counteract against it, they can move very elegant in smooth waveforms over the ground. Scientists have been very fascinated by this form of movement for a long time und try to simulate this smooth kind of movement. In general it could not be found out how the animals move that elegant on smooth surfaces. On rough surfaces, this ability goes back on their scalelike body. In Germany there are only two poisonous kinds of snakes: the common viper and the asp viber. Both kinds are strictly protected animals by nature conservation and are listed on the red list as threatened to extinction. Because of the rarity of the animals bite accidents are relatively rare. But in the last 60 years, there was no single case of death in the last 60 years related to a snake bite. The viper (Colubridae) has no poisonous fangs and is harmless. The viper snakes differ from the native poisonous snakes regarding their round pupils. Snakes have always been fascinating to humans. Because of that, they play a big role in cultural history and mythology. For example in ancient Greece, snakes were considered as sacred. Why? Because of the fact that snakes often peel off their skin, humans mad them to a symbol of re-renewal and immortality.

Furthermore, snakes were thought to have healing powers. This also leaded to the snake being a symbol for the status of doctors. Unit today the snake is still in the sign of the staff of Aesculapius, which can be found in simplified form in some signs of pharmacies. Snakes were also suggested to have the ability of clairvoyance, therefore they were the animals of the goddess Gaia. According to Hesiod Gaia Pelope was one of the many names of the earth goddess Gaia. In the Oracle of Delphi snake priests (Pythea) were in service of Gaia. Not only in the christian-jewish tradition existed a tree protected by snakes. In the ancient Greek imagination there was a life spending apple tree in the garden of the Hesperides, which is guarded by the snake Ladon and was given as a present to the goddess Hera by Gaia.

1.2.2 Multimodal Sensory intervention (MMSS).

The visual stimulation comprised toneless video trailers of the following fantasy movies: “The Hobbit” (Peter Jackson, Warner Home Video), “Avatar” (James Cameron, 20th Century Fox Home Entertainment), “Wizard of Oz” (Sam Raimi, Walt Disney), “Pans Labyrinth” (Javier Navarrete, Universum Film GmbH), “Epic” (Danny Elfman, 20th Century Fox Home Entertainment), and “Jack and the Giants” (Bryan Singer, Warner Home Video). At the same time, subjects listen binaurally via headphones to music by Fred Frith (e.g. “Voice of America part 3/ LEGS” by Fred Frith, Step across the border, RecRec Music). Individuals were further sitting on a massage chair, which provided haptic stimulation by a intensive massage of the back area (Medisana MCN Shiatsu massage seat, program: whole back).

1.3 Experimental Procedure

1.3.1 Stimuli Characteristics

Conditioned stimuli (CS). Two fear-relevant stimuli were used as CS+ (spider and snake, IAPS numbers 1220 and 1052), as well as a fear-irrelevant stimulus as CS- (mug, IAPS number 7009) [7]. Analogous to Kindt and colleagues [8, 9], we chose fear-relevant stimuli, since these have been shown to be particularly resistant to extinction learning and ecologically valid, as they have been linked to fear responses in clinical samples (see also [10]. One of the two CS+ (CS+R) was reactivated during the memory reactivation phase on day 2 while the other served as a control CS+ (CS+NR). Assignment of the pictures as CS+R and CS+NR was counterbalanced across participants. The fear-irrelevant stimulus (CS-) was never associated with an aversive event. The CS- provides a baseline since it should not elicit a fear response. All pictures were presented at the center of a 17'' computer screen (resolution 1024 x 786 x 32 pixel, picture size 600 x 450 pixel).

Unconditioned stimulus (US): As US an electric stimulation of 2 ms was applied to the wrist of the non-preferred arm. Delivery of electric stimulation was controlled by the Digitimer DS7A constant current stimulator (Digitimer, Herfordshire, UK) via a bar stimulating electrode with two durable stainless steel disk electrodes (8 mm diameter, distance 30 mm) placed on the upper wrist of the non-preferred hand and fixated with a Velcro strap. Electrodes were filled with conductive gel (Signa Gel, Parker). Shock intensity was determined individually for each participant on day 1 before the start of testing. Starting at an intensity of 1 mA, the electric stimulus was delivered to the non-preferred hand and gradually increased (2mA steps), until subject rated it as "very unpleasant, but not painful" (see e.g. [11, 12]. Participants were instructed that the individually selected intensity remained set during all three days.

Startle probe. The startle probe consisted of a burst of white noise (40 ms, 95 dB, bandwith of 20 Hz – 20 kHz) and was presented during each CS presentation, as well as during habituation and noise alone trials, binaurally via headphones (Sennheiser HD 25-1-II).

2. Results

2.1 Fear Acquisition

US Online Expectancy Ratings (OE-R)

RA compared to Placebo: Comparing RA to placebo revealed similar effects as the analyses of propranolol and placebo: OE-R differed between stimulus types depending on time (stimulus x time: $F(6,216) = 77.51$, $p < .001$; see Table 2, Fig 2) with no influence of the intervention condition (intervention x stimulus x time: $F(6,216) = 1.02$, $p = .414$). OE-R change could be confirmed for both CS+ as compared to the CS- (post hoc contrasts: CS+R: $F(1,36) = 158.07$, $p < .001$; CS+NR: $F(1,36) = 207.48$, $p < .001$). No differences were confirmed between both CS+ (2x4-ANOVA sub-design: stimulus x time: $F(3,111) = 1.72$, $p=.168$). Post-hoc tests revealed higher OE-R to both CS+ as compared to the CS- at the end of fear learning (both $p<.001$), while no differences were confirmed between both CS+ ($p > .1$).

MMSS compared to Placebo: OE-R differed between stimulus types depending on time ($F(6,222) = 68.56$, $p < .001$; see Table 2, Fig 2) without an influence of the intervention conditions (intervention x stimulus x time: $F(6,222) = .21$, $p = .975$). OE-R change differed between all three stimulus types (post-hoc contrasts: CS+R: $F(1,36) = 128.85$, $p < .001$; CS+NR: $F(1,36) = 210.49$, $p < .001$; 2x4-ANOVA sub-design with CS+R and CS+NR: stimulus x time: $F(3,114) = 4.37$, $p = .006$; linear contrast: $F(1,37) = 13.14$, $p < .001$). At the end of the acquisition phase, post-hoc tests revealed higher OE-R to both CS+ as compared to the CS- (both $p < .001$) and no differences between both CS+ ($p > .1$). In addition, OE-R

differed as a trend between intervention conditions depending on the stimulus type (intervention x stimulus: F(2,74) = 3.12, *p* = .050; post-hoc linear trend: CS+R: F(1,36) = 5.46, *p* = .025; CS+NR: F(1,36) = 2.57, *p* = .117; 2x2x4-ANOVA sub-design with CS+R and CS+NR: intervention x stimulus: F(1,37) = .19, *p* = .664) and time (; (intervention x time: (F(3,111) = 2.64, *p* = .053, post-hoc linear trend: F(1,37) = 3.63, *p* = .064): OE-R to the CS- in the placebo was lower as compared to MMSS condition (post hoc: *p* = .040, both other *p* > .1).

Fear Potentiated Startle (FPS)

RA compared to Placebo: The comparison of RA and placebo revealed similar results as the analysis contrasting the propranolol intervention with placebo (see Table 3, Fig 2): FPS differed between stimulus types depending on time without an influence of the intervention condition (stimulus x time: F(6,222) = 2.99, *p* = .008; intervention x stimulus x time: F(6,222) = 0.71, *p* = .637). The linear trend over time differed for both the CS+R and the CS+NR compared to the CS- (post-hoc contrasts: CS+R: F(1,36) = 7.94, *p* = .008; CS+NR: F(1,36) = 7.86, *p* = .008). A direct comparison of FPS between both CS+ revealed no differences in FPS between these stimulus types over the course of the acquisition phase (2x4-ANOVA sub-design: stimulus x time: F(3,111) = 1.31, *p* = .274). At the end of the acquisition phase, FPS was higher to both CS+ as compared to the CS- (both *p* < .001), but not significantly different between both CS+ (*p* = 0.557).

MMSS compared to Placebo: Statistical analyses revealed a change of FPS over time that was different between stimulus types statistically only as a trend (F(6,222) = 1.88, *p* = .085; intervention x stimulus x time: F(6,222) = 1.53, *p* = .169; see Table 3, Fig 2). The linear trend over time differed for the CS+NR, but not for the CS+R as compared to the CS- (post-hoc contrasts: CS+R: F(1,36) = 2.11, *p* = .154; CS+NR: F(1,36) = 5.83, *p* = .021). However, a direct comparison of FPS between both CS+ revealed no differences in FPS between these

stimulus types over the course of the acquisition phase (2x4-ANOVA sub-design: stimulus x time: F(3,111) = 2.06, p = .110). Nevertheless, at the end of the acquisition phase, FPS was higher to both CS+ as compared to the CS- (both p < .005), but not significantly different between both CS+ (p = .395).

2.2 Retention

US Expectancy Ratings

RA compared to Placebo: Comparing RA to placebo revealed similar effects as the analyses of propranolol and placebo: OE-R differed between stimulus types depending on time without a modulating effect of the intervention condition (F(2,74) = 7.24, p = .001; intervention x stimulus x time: F(2,74) = 2.03, p = .139; see Table 2, Fig 2). OE-R change could be confirmed to both CS+ as compared to the CS- (post-hoc contrasts: all p's < .003), while no differences were confirmed between both CS+ (2x2-ANOVA sub-design: stimulus x time: F(1,38)< .01, p > .1). Post-hoc tests revealed a reduction of OE-R for both CS+ from end of acquisition to the start of the extinction phase (both p < .005), while there was no change in OE-R for CS- (p = .458). OE-R was higher in response to both the CS+R and CS+NR compared to the CS- (both p < .001) without a difference between OE-R of both CS+ (p > .1) at the start of the extinction phase.

MMSS compared to Placebo: OE-R differed between stimulus types depending on time without an influence of the intervention condition (F(2,74) = 8.15, p = .001; (intervention x stimulus x time: F(2,74) = .21, p = .815; see Table 2, Fig 2). The OE-R change from end of the acquisition to start of extinction phase differed for the CS+R as compared to the CS- (post hoc contrast: p < .001). However, OE-R change for the CS+NR differed from CS- only as a trend (post hoc contrast: p = .085) and was less pronounced than OE-R change to the CS+R (2x2-ANOVA sub-design: stimulus x time: F(1,38) = 4.63, p = .038). Post-hoc tests revealed

lower OE-R to the CS+NR during retention testing as compared to the end of fear learning (p = .001), while no differences were confirmed for CS+R and CS- between phases (p > .156). However, OE-R to both CS+ was higher than to the CS- at the start of the extinction phase (p<.001), without a difference between OE-R of both CS+ (p > .1).

Fear Potentiated Startle

RA compared to Placebo: FPS increased over time (F(1,37) = 15.94, p < .001; see Table 3, Fig 2). However, this change was neither influenced by the stimulus type, nor by intervention condition (stimulus x time: F(2,74) = 0.62, p = .538, intervention x stimulus x time: F(2,74) = 1.24, p = .297). In general, FPS was higher to both CS+ as compared to the CS-, with no differences between both CS+ (stimulus: F(2,74) = 12.89, p <.001; post-hoc tests: CS+R vs. CS-: p = .002; CS+NR vs. CS-: p <.001; CS+R vs. CS+NR: p > .1).

MMSS compared to Placebo: Comparing MMSS to placebo revealed similar effects as the analyses of RA and placebo: FPS increased over time (time: F(1,37) = 24.97, p < .001; see Table 3, Fig 2) without an influence of the stimulus type or the intervention condition (stimulus x time: (F2,74) = .221, p = .814; intervention x stimulus x time: F(2,74) = 1.68, p=.194). In general, FPS was higher to both CS+ as compared to the CS-, with no differences between both CS+ (stimulus: F(2,74) = 9.71, p < .001; post-hoc tests: CS+R vs. CS-: p = .005; CS+NR vs. CS-: p = .001; CS+R vs. CS+NR: p > .1).

<u>2.3 Extinction learning</u>

US-Expectancy Ratings.

RA compared to Placebo: Comparing RA to placebo revealed similar effects as the analyses of propranolol and placebo: OE-R differed between stimulus types depending on time (F(2,74) = 179.13, p < .001; see Table 2, Fig 2), without an influence of the intervention condition (intervention x stimulus x time: F(2,74) = .14, p = .870). OE-R change differed for

both CS+ as compared to the CS- (post hoc contrasts: all *p*'s < .001), while there was no difference between OE-R change between both CS+ (2x2-ANOVA sub-design: stimulus x time: F(1,38) = .38, *p* = .540). Post-hoc tests revealed lower OE-R to both CS+ during the end as compared to the beginning of extinction learning (both *p* < .001), while no differences were confirmed for CS- between phases (*p* = .153). At the end of the extinction phase, OE-R was still higher to both CS+ compared to the CS- (both *p* < .005).

MMSS compared to Placebo: Comparing MMSS to placebo revealed similar effects as the analyses of the propranolol and RA interventions compared to placebo: OE-R differed between stimulus types depending on time (F(2,74) = 116.43, *p* < .001; see Table 2, Fig 2), but independent of the intervention condition (intervention x stimulus x time: F(2,74) = 1.86, *p*=.164). OE-R change differed to both CS+ as compared to the CS- from the beginning to the end of the extinction phase (post-hoc contrasts: all *p*'s <.001), while no differences were confirmed in OE-R change between both CS+ (2x2-ANOVA sub-design: stimulus x time: F(1,38) = .53, *p* = .473). Post-hoc tests revealed lower OE-R to both CS+ during the end as compared to the beginning of extinction learning (both *p* < .001), while OE-R decreased only as a trend for CS- between phases (*p* = .092). At the end of the extinction phase, OE-R was still higher to both CS+ compared to the CS- (both *p* < .001).

Fear potentiated startle

RA compared to Placebo: FPS decreased over time (F(1,36) = 96.77, *p* <.001; see Table 3, Fig 2) independent of the intervention condition and the stimulus type (intervention x stimulus x time: F(2,72) = .56, *p* = .576; stimulus x time: F(2,72) = .99, *p* = .376). However, FPS differed between intervention conditions depending on the stimulus types (intervention x stimulus: F(2,72) = 3.56, *p* = .040). While pairwise comparison of the intervention conditions for the stimulus types revealed, as a trend, only a higher FPS to CS+R during placebo compared to RA (*p* = .053), an explorative analyses of mean FPS suggests that this difference

may be attributable particularly to differences at the end of the extinction phase (start extinction: $p = .072$, end extinction $p = .020$).

MMSS compared to Placebo: FPS decreased over time ($F(1,36) = 125.84$, $p <.001$; see Table 3, Fig 2), without an influence of the intervention condition or the stimulus type (intervention x stimulus x time: $F(2,72) = 1.77$, $p = .177$; stimulus x time: $F(2,72) = 1.69$, $p = .192$). In general, FPS was higher to the CS+R as compared to the CS-, with no differences between CS+NR and CS- or both CS+ (stimulus: $F(2,72) = 5.03$, $p < .001$; post-hoc tests: CS+R vs. CS-: $p = .012$; CS+NR vs. CS- $p = .120$; CS+R vs. CS+NR: $p > .1$).

2.4 Reinstatement

US Expectancy Ratings

RA compared to Placebo: OE-R differed between stimulus types depending on time ($F(2,70) = 6.54$, $p = .002$; see Table 2, Fig 2). While OE-R change over time differed for both CS+ compared to the CS- (post hoc contrasts: all *p*'s < .024), it was not distinguishable between both CS+ (2x2-ANOVA sub-design: stimulus by time: $F(1,36) = 1.12$, $p = .275$). In general, OE-R decreased stronger in the RA than in the placebo condition (intervention x time: $F(1,35) = 4.66$, $p = .038$). At reinstatement testing, OE-R was higher to both CS+ compared to the CS- (both $p < .009$), with no difference between both CS+ ($p > .1$).

MMSS compared to Placebo: Comparing MMSS to placebo revealed similar effects as the analyses of propranolol and placebo: OE-R differed between stimulus types depending on time independent of the intervention condition (stimulus x time: $F(2,74) = 10.65$, $p <. 001$; intervention x stimulus x time: $F(2,74) = .178$, $p = .837$; see Table 2, Fig 2). The OE-R change from the end of the extinction phase to reinstatement differed for both the CS+R and the CS+NR as compared to the CS- (post hoc contrasts: $p < .001$), while there was no difference in OE-R between the two CS+ (2x2 ANOVA sub-design: stimulus x time: F(1,38)

= 1.75, p = .193). At reinstatement testing, OE-R was higher to both CS+ compared to the CS- (both $p < .001$), with no difference between both CS+ ($p > .1$).

Fear Potentiated Startle.

RA compared to Placebo: Comparing RA to placebo revealed similar effects as the analyses of propranolol and placebo: FPS increased over time (F(1,36) = 24.48, $p < .001$; see Table 3, Fig 2), independent of the intervention condition and stimulus type (intervention x stimulus x time: F(2,72) = .23, p = .793; stimulus x time: F(2,72) = .25, p = .776). In general, FPS differed between stimulus types (F(2,72) = 10.88, $p < .001$). Post hoc tests revealed a larger FPS to both CS+ as compared to the CS- overall (CS+R vs. CS-: p = .002; CS+NR vs. CS-: $p < .001$), with no differences between CS+ ($p > .1$).

MMSS compared to Placebo: Comparing MMSS to placebo revealed similar effects as the analyses of the other intervention conditions: FPS increased over time (F(1,36) = 21.49, $p < .001$; see Table 3, Fig 2) independent of the intervention condition and stimulus type (intervention x stimulus x time: F(2,72) = 34, p = .713; stimulus x time: F(2,72) = 2.05, p = .136). In general, FPS differed between stimulus types (F(2,72 = 4.46, p = .015). Post hoc tests revealed a larger FPS to the CS+R as compared to the CS- (p = .043), a marginal significant effect comparing CS+NR to CS- (p = .061) and no differences between both CS+ ($p > .1$).

2.5 Prediction error

To assess the degree of a prediction error on day 2 [13, 14], a 2 x 2 rmANOVA with the between-subjects factor ‘type of intervention’ and the within-subject factor ‘time’ (end of acquisition, reactivation trial).

2.5.1 US – Expectancy (OE-R)

Propranolol compared to Placebo: OE-R did not differ between the end of acquisition and reactivation ($F(1,36) = .31$, $p = .583$), and there was no influence of the intervention conditions (intervention x time: $F(1,36) = .63$, $p = .433$).

RA compared to Placebo: Comparing RA to placebo revealed similar effects as the analyses of propranolol and placebo: OE-R did not differ between the end of acquisition and reactivation ($F(1,36) = .28$, $p = .599$), and there was no influence of the intervention conditions (intervention x time: $F(1,36) = .07$, $p = .796$).

MMSS compared to Placebo: Comparing MMSS to placebo revealed similar effects as the analyses of propranolol and placebo: OE-R did not differ between the end of acquisition and reactivation ($F(1,35) = .01$, $p = .908$), and there was no influence of the intervention conditions (intervention x time: $F(1,36) = .14$, $p = .708$).

<u>*2.5.2 Fear potentiated startle (FPS)*</u>

Propranolol compared to Placebo: FPS increased over time ($F(1,35) = 13.07$, $p = .001$), while this effect was independent of the intervention condition (intervention x time: $F(1,35) <.001$, $p = .996$).

RA compared to Placebo: Comparing RA to placebo revealed similar effects as the analyses of propranolol and placebo: FPS increased over time ($F(1,36) = 16.26$, $p < .001$), while this effect was independent of the intervention condition (intervention x time: $F(1,36) = .09$, $p = .773$).

MMSS compared to Placebo: Comparing MMSS to placebo revealed similar effects as the analyses of propranolol and placebo: FPS increased over time ($F(1,34) = 18.24$, $p < .001$), while this effect was independent of the intervention condition (intervention x time: $F(1,34) = .92$, $p = .344$).

2.6 Trait anxiety and effects of propranolol on fear memory reconsolidation

2.6.1 FPS Retention analyses controlled for trait anxiety

To assess, whether trait anxiety might impact the observed effects within the propranolol intervention condition, we reran the analyses with trait anxiety as a covariate. FPS still differed between the two intervention conditions depending on stimulus type and time (intervention x stimulus x time: $F(2,70) = 5.71$, $p = .005$). Intervention conditions differed in FPS change over time when comparing the CS+NR to the CS- ($F(1,35) = 8.51$, $p = .006$), but not when comparing the CS+R to the CS- ($F(1,35) = .095$, $p = .759$). A direct comparison of both CS+ with an additional ANOVA sub-design revealed that intervention conditions differed in FPS change also for CS+R and CS+NR (2x2x2-ANOVA sub-design: intervention x stimulus x time: $F(1,35) = 8.00$, $p = .008$). At the start of the extinction phase, FPS was higher in response to the CS+NR as compared to both the CS- as well as to the CS+R in the propanol condition (both p <. 005), while FPS did not differ between the three stimulus types in the placebo condition (all $p > .684$). Thus, controlling for trait anxiety supports the observed effects.

2.7 Generalization to the control stimulus

To test whether the observed generalization to the control stimulus during retention and reinstatement testing was associated with trait anxiety, we run Pearson correlations between a) the change in FPS of the CS- regarding retention testing (CS- start extinction minus CS- end acquisition) and b) reinstatement testing (CS- end extinction minus CS- reinstatement trial) overall subjects. We did not observe a significant association (Retention: $r = .01$, $p = .928$; Reinstatement; $r = -.05$, $p = .685$).

References

1. Laux L, Glanzmann P, Schaffner P, Spielberger CD. Das State-Trait- Angstinventar. Theoretische Grundlagen und Handanweisung. Weinheim: Beltz; 1981.

2. Spielberger CD, Sydemann SJ, Owen AE, Marsh BJ. Measuring anxiety and anger with the State-Trait Anxiety Inventory (STAI) and the State-Trait Anger Expression Inventory (STAXI). In: Maruish ME, editor. Then use of psychological testing for treatment planning and outcomes assessment. 2. Mahwah, NJ: Erlbaum; 1999. p. 993-1021.

3. Kabacoff RI, Segal DL, Hersen M, Van Hasselt VB. Psychometric properties and diagnostic utility of the Beck Anxiety Inventory and the State-Trait Anxiety Inventory with older adult psychiatric outpatients. Journal of anxiety disorders. 1997;11(1):33-47. Epub 1997/01/01. PubMed PMID: 9131880.

4. Rinck M, Bundschuh S, Engler S, Müller A, Wissmann J, Ellwart T, et al. Reliabilität und Validität dreier Instrumente zur Messung von Angst vor Spinnen. Diagnostica. 2002;48:141-9.

5. Klorman R, Hastings JE, Weerts TC, Melamed BG, Lang PJ. Pyschometric description of some specific-fear questionnaires Behavior Therapy. 1974;5:401-9.

6. Klieger DM. The snake anxiety questionnaire as a measure of ophidophobia. Educational and Psychological Measurement. 1987;47:449-59.

7. Lang PJ, Bradley MM, Cuthbert BN. International Affective Picture System (IAPS): Affective Ratings of Pictures and Instruction Manual Gainesville: University of Florida; 2008.

8. Soeter M, Kindt M. Erasing fear for an imagined threat event. Psychoneuroendocrinology. 2012;37(11):1769-79. Epub 2012/04/17. doi: 10.1016/j.psyneuen.2012.03.011. PubMed PMID: 22503387.

9. Soeter M, Kindt M. Disrupting reconsolidation: pharmacological and behavioral manipulations. Learning & memory (Cold Spring Harbor, NY). 2011;18(6):357-66. Epub 2011/05/18. doi: 10.1101/lm.2148511. PubMed PMID: 21576515.

10. Ohman A, Mineka S. Fears, phobias, and preparedness: toward an evolved module of fear and fear learning. Psychol Rev. 2001;108(3):483-522. Epub 2001/08/08. PubMed PMID: 11488376.

11. Soeter M, Kindt M. Dissociating response systems: erasing fear from memory. Neurobiology of learning and memory. 2010;94(1):30-41. Epub 2010/04/13. doi: 10.1016/j.nlm.2010.03.004. PubMed PMID: 20381628.

12. Soeter M, Kindt M. Stimulation of the noradrenergic system during memory formation impairs extinction learning but not the disruption of reconsolidation. Neuropsychopharmacology : official publication of the American College of Neuropsychopharmacology. 2012;37(5):1204-15. Epub 2011/12/16. doi: 10.1038/npp.2011.307. PubMed PMID: 22169947; PubMed Central PMCID: PMCPmc3306881.

13. Sevenster D, Beckers T, Kindt M. Prediction error demarcates the transition from retrieval, to reconsolidation, to new learning. Learning & memory (Cold Spring Harbor, NY). 2014;21(11):580-4. Epub 2014/10/17. doi: 10.1101/lm.035493.114. PubMed PMID: 25320349; PubMed Central PMCID: PMCPmc4201815.

14. Sevenster D, Beckers T, Kindt M. Prediction error governs pharmacologically induced amnesia for learned fear. Science (New York, NY). 2013;339(6121):830-3. Epub 2013/02/16. doi: 10.1126/science.1231357. PubMed PMID: 23413355.
