# Supplementary figures and images for: Modification of Fear Memory by Pharmacological and Behavioural Interventions during Reconsolidation
Source: PLoS One. 2016 Aug 18;11(8):e0161044. doi: 10.1371/journal.pone.0161044 (PMC4990323; doi:10.1371/journal.pone.0161044)

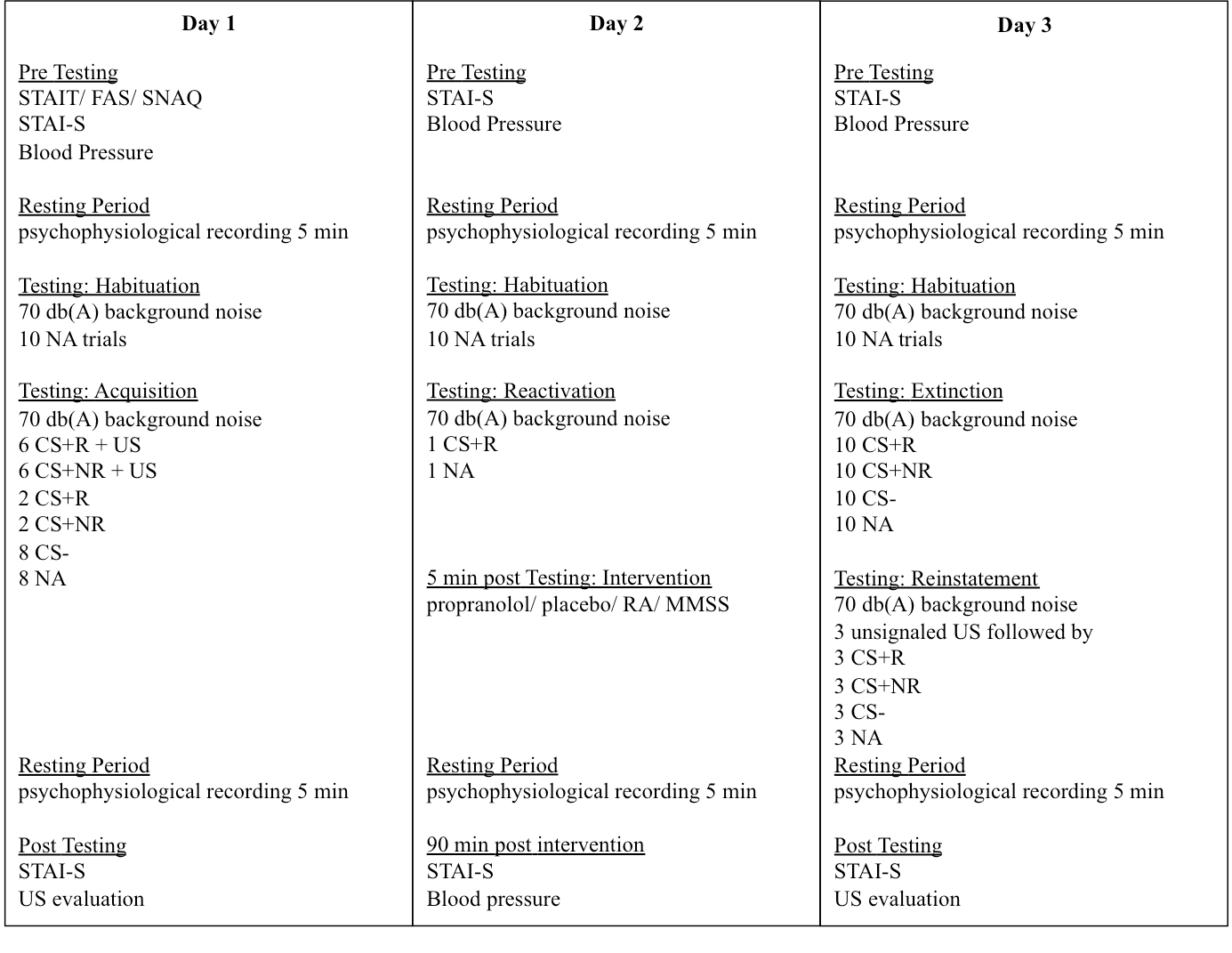

Supplement: S1 Fig — Detailed description of the experimental procedure over three consecutive days. (TIF) [file pone.0161044.s002.tif]
