# Supplementary material for: Modification of Fear Memory by Pharmacological and Behavioural Interventions during Reconsolidation
Source: PLoS One. 2016 Aug 18;11(8):e0161044. doi: 10.1371/journal.pone.0161044 (PMC4990323; doi:10.1371/journal.pone.0161044)
Supplement: S1 Table — Mean values (SD) of systolic and diastolic blood pressure (in mmHg) and HR pre and 90 minutes post propranolol administration during memory reactivation on day 2. (PDF) [file pone.0161044.s003.pdf]

Supplemental Information

S1 Table. Systolic, and diastolic blood pressure, and heart rate.

Mean values (SD) of systolic and diastolic blood pressure (in mmHg) and HR pre and 90 minutes post propranolol administration during memory reactivation on day 2.

		Pre-pill intake	Post-pill intake
Placebo			
	Systolic BP	109.16 (13.15)	111.68 (14.02)
	Diastolic BP	66.21 (6.47)	69.16 (10.99)
	HR	77.89 (12.17)	67.89 (10.47)
Propranolol			
	Systolic BP	108.16 (12.79)	106.47 (13.62)
	Diastolic BP	65.94 (7.02)	68.73 (12.38)
	HR	79.89 (12.27)	64.37 (8.19)
